# Regulation of *NCAPG* by *miR‐99a‐3p* (passenger strand) inhibits cancer cell aggressiveness and is involved in CRPC

**DOI:** 10.1002/cam4.1455

**Published:** 2018-04-02

**Authors:** Takayuki Arai, Atsushi Okato, Yasutaka Yamada, Sho Sugawara, Akira Kurozumi, Satoko Kojima, Kazuto Yamazaki, Yukio Naya, Tomohiko Ichikawa, Naohiko Seki

**Affiliations:** ^1^ Department of Functional Genomics Chiba University Graduate School of Medicine Chiba Japan; ^2^ Department of Urology Chiba University Graduate School of Medicine Chiba Japan; ^3^ Department of Urology Teikyo University Chiba Medical Center Ichihara Japan; ^4^ Department of Pathology Teikyo University Chiba Medical Center Ichihara Japan

**Keywords:** Castration‐resistant prostate cancer, microRNA, *miR‐99a‐3p*, *miR‐99a‐5p*, non‐SMC condensin I complex subunit G

## Abstract

Effective treatments for patients with castration‐resistant prostate cancer (CRPC) have not yet been established. Novel approaches for identification of putative therapeutic targets for CRPC are needed. Analyses of RNA sequencing of microRNA (miRNA) expression revealed that *miR‐99a‐3p* (passenger strand) is significantly downregulated in several types of cancers. Here, we aimed to identify novel *miR‐99a‐3p* regulatory networks and therapeutic targets for CRPC. Ectopic expression of *miR‐99a‐3p* significantly inhibited cancer cell proliferation, migration, and invasion in PCa cells. Non‐SMC condensin I complex subunit G (*NCAPG*) was a direct target of *miR‐99a‐3p* in PCa cells. Overexpression of NCAPG was detected in CRPC clinical specimens and was significantly associated with shorter disease‐free survival and advanced clinical stage. Knockdown of *NCAPG* inhibited cancer cell aggressiveness. The passenger strand *miR‐99a‐3p* acted as an antitumor miRNA in naïve PCa and CRPC. *NCAPG* was regulated by *miR‐99a‐3p*, and its overexpression was involved in CRPC pathogenesis. Involvement of passenger strand of miRNA in cancer pathogenesis is novel concept, and identification of antitumor miRNA regulatory networks in CRPC might be provided novel prognostic markers and therapeutic targets for this disease.

## Introduction

In developed countries, prostate cancer (PCa) is one of the most commonly diagnosed cancers, identified by prostate‐specific antigen (PSA) screening; PCa is also the third leading cause of cancer‐related death among men 1. Most naïve PCa initially responds well to androgen‐deprivation therapy (ADT). However, during ADT treatment, PCa cells acquire ADT treatment resistance and progress to a lethal pathology known as castration‐resistant prostate cancer (CRPC) 2. Cancer cells that have reached CRPC can cause distant metastasis, and effective treatments for patients with CRPC have not yet been established 3. Identification of the molecular pathogenesis underlying acquisition of androgen‐independent and metastatic signaling pathways based on advanced genomic approaches is essential for further understanding of this disease.

MicroRNAs (miRNAs) are endogenous small RNAs (molecules 18–23 bases in length) that act as central players regulating the expression control of protein‐coding and protein‐noncoding RNAs 4, 5. Interestingly, a single miRNA can directly regulate a vast number of RNAs in human cells 6. Therefore, aberrant expression of miRNAs can disrupt normal control of RNA expression in cancer cells. Furthermore, dysregulation of miRNAs is contributed to cancer cell malignancies, such as progression, metastasis, and treatment resistance 7, 8, 9, 10.

Analysis of our original miRNA expression signatures of cancers based on RNA sequencing revealed that several passenger strands of miRNAs, for example, *miR‐145‐3p*,* miR‐150‐3p*,* miR‐149‐3p*,* miR‐199a‐3p*, and *miR‐144‐5p*, are downregulated in several cancer tissues and act as antitumor miRNAs in cancer cells 11, 12, 13, 14, 15. However, this is inconsistent with the paradigm that the guide strand of miRNA is loaded into the miRNA‐induced silencing complex (RISC) and represses translation or degradation of target genes 16, whereas the passenger strand of miRNA is thought to be destroyed in the cytoplasm and to have no function 17, 18, 19.

We have sequentially identified the functional significance of passenger strands of miRNAs in cancer cells based on miRNA signatures 11, 12, 13, 14, 15. In this study, we focused on *miR‐99a‐5p* (guide strand) whose expression was significantly downregulated in our miRNA signature of metastatic CRPC 15 and investigated the functional roles including passenger strand *miR‐99a‐3p* in naïve PCa and CRPC cells. Previous studies have shown that the guide strand *miR‐99a‐5p* has antitumor roles in several cancers 20, 21, 22, 23. In contrast, no studies have reported the role of the passenger strand *miR‐99a‐3p* in cancer cells. Novel strategies based on passenger strands of miRNAs will enhance our understanding of the molecular pathways underlying naïve PCa and CRPC pathogenesis.

## Materials and Methods

### Collection of clinical prostate specimens and cell lines

Clinical specimens were collected at Teikyo University Chiba Medical Center and Chiba University Hospital from 2013 to 2016. Patient characteristics and clinical features are summarized in Table 1. The protocol of this study was approved by the Institutional Review Boards of Teikyo University and Chiba University. We have experimented with human PCa cell lines (PC3, DU145, and C4‐2). The cells were maintained as previously reported 11, 15, 24, 25.

**Table 1:** Patient characteristics

Patient No.	Procedure	Diagnosis	Age (years)	PSA (ng/mL)	Gleason score	*T*	*N*	*M*	Stage	Remarks
1	Biopsy	Non‐PCa	57	5.71	–	–	–	–	–	RT‐PCR
2	Biopsy	Non‐PCa	74	9.45	–	–	–	–	–	RT‐PCR
3	Biopsy	Non‐PCa	70	8.58	–	–	–	–	–	RT‐PCR
4	Biopsy	Non‐PCa	73	4.8	–	–	–	–	–	RT‐PCR
5	Biopsy	Non‐PCa	67	6.91	–	–	–	–	–	RT‐PCR
6	Biopsy	Non‐PCa	50	7.05	–	–	–	–	–	RT‐PCR
7	Biopsy	Non‐PCa	74	9.91	–	–	–	–	–	RT‐PCR
8	Biopsy	Non‐PCa	76	20.9	–	–	–	–	–	RT‐PCR
9	Biopsy	Non‐PCa	59	4.5	–	–	–	–	–	RT‐PCR
10	Biopsy	Non‐PCa	75	1.1	–	–	–	–	–	RT‐PCR
11	Biopsy	Non‐PCa	60	7.29	–	–	–	–	–	RT‐PCR
12	Biopsy	Non‐PCa	73	38.7	–	–	–	–	–	RT‐PCR
13	Biopsy	Non‐PCa	69	11.9	–	–	–	–	–	RT‐PCR
14	Biopsy	Non‐PCa	77	23.3	–	–	–	–	–	RT‐PCR
15	Biopsy	Non‐PCa	61	4.57	–	–	–	–	–	RT‐PCR
16	Biopsy	Non‐PCa	59	7.37	–	–	–	–	–	RT‐PCR
17	Biopsy	Non‐PCa	65	5.06	–	–	–	–	–	RT‐PCR
18	Biopsy	HSPC	70	75.7	4 + 5	4	1	1	IV	RT‐PCR
19	Biopsy	HSPC	78	1800	4 + 5	4	1	1	IV	RT‐PCR
20	Biopsy	HSPC	75	68.4	5 + 4	4	1	0	IV	RT‐PCR
21	Biopsy	HSPC	62	38.7	4 + 5	2b	1	0	IV	RT‐PCR
22	Biopsy	HSPC	70	25.5	4 + 5	3b	0	0	III	RT‐PCR
23	Biopsy	HSPC	88	888	4 + 5	3b	1	1	IV	RT‐PCR
24	Biopsy	HSPC	69	33.9	4 + 5	4	0	1	IV	RT‐PCR
25	Biopsy	HSPC	62	62.3	4 + 5	3b	1	0	IV	RT‐PCR
26	Biopsy	HSPC	78	5	4 + 5	2c	0	1b	IV	RT‐PCR
27	Biopsy	HSPC	64	449	4 + 5	3b	1	1	IV	RT‐PCR
28	Biopsy	HSPC	81	365	4 + 5	4	1	1	IV	RT‐PCR
29	Biopsy	HSPC	76	715	5 + 4	4	1	1	IV	RT‐PCR
30	Biopsy	HSPC	79	555	4 + 5	3	1	1	IV	RT‐PCR
31	Biopsy	HSPC	63	1120	4 + 5	2c	0	1b	IV	RT‐PCR
32	Biopsy	HSPC	67	4.95	4 + 5	4	1	1b	IV	RT‐PCR
33	Biopsy	HSPC	70	19.5	5 + 5	4	1	1c	IV	RT‐PCR
34	Biopsy	CRPC	69	15.8	5 + 4	3b	1	1	IV	RT‐PCR
35	Biopsy	CRPC	72	212	5 + 4	4	1	1	IV	RT‐PCR
36	Biopsy	CRPC	71	4.4	4 + 5	4	1	1	IV	RT‐PCR
37	Biopsy	CRPC	68	7.54	4 + 5	4	1	1b	IV	RT‐PCR
38	Prostatectomy	HSPC	65	5.3	4 + 5	2a	0	0	II	IHC
39	Prostatectomy	HSPC	61	21.48	4 + 4	3a	0	0	III	IHC
40	Autopsy	CRPC	64	4100	4 + 5	4	1	1c	IV	IHC
41	Autopsy	CRPC	75	4690	4 + 5	4	1	1c	IV	IHC

### Quantitative real‐time reverse transcription polymerase chain reaction (qRT‐PCR)

The procedure of PCR quantification is described in our previous reports 11, 15, 24, 25, 26. Expression levels of *miR‐99a‐5p* and *miR‐99a‐3p* normalized to expression of *RNU4*8 were analyzed by TaqMan qRT‐PCR. The expression levels of *NCAPG* and pri‐*miR‐99a* were assessed by being normalized with *GAPDH* or *GUSB*. Detailed product numbers of reagents used are shown in the Table S1.

### Transfection with mature miRNA, small‐interfering RNA (siRNA), or plasmid vectors

We used the mature miRNAs, siRNAs, and plasmid vectors described below: Pre‐miR miRNA precursor (*hsa‐miR‐99a‐5p*; assay ID: PM10719 and *hsa‐miR‐99a‐3p*; assay ID: PM12983; Applied Biosystems, Foster City, CA), Stealth Select RNAi siRNAs; si‐*NCAPG* (cat. nos. HSS127430 and HSS184671; Invitrogen, Carlsbad, CA), and negative control miRNA/siRNA (P/N: AM17111; Applied Biosystems). RNAs were incubated with OPTI‐MEM (Invitrogen) and Lipofectamine RNAiMax reagent (Invitrogen) at a concentration of 10 nmol/L by reverse transfection. We used *NCAPG* plasmid vector designed by ORIGENE (cat. no. SC111395; Rockville, MD). Transfection procedures were described as previous studies 11, 15, 24, 25, 26.

### Cell proliferation, migration, and invasion assays

As functional analyses, cell proliferation, migration, and invasion assays were carried out based on our past reports 11, 15, 24, 25, 26. We confirmed all experiments in triplicate.

### Confirmation of miRNAs incorporated into the RNA‐induced silencing complex (RISC) by Ago2 immunoprecipitation

To investigate whether exogenous *miR‐99a‐5p* and *miR‐99a‐3p* were incorporated into the RISC, we carried out immunoprecipitation assays using a microRNA isolation kit for human Ago2 (Wako, Osaka, Japan). The procedure is described in our past reports 11, 15.

### Identification strategy of estimated target genes regulated by *miR‐99a‐3p* in PCa cells

To identify putative *miR‐99a‐3p* target genes, we used in silico database analyses and comprehensive gene expression analyses by microarray technologies, as described previously 11, 15, 24, 25, 26. The microarray data were deposited into the GEO database (https://www.ncbi.nlm.nih.gov/geo/; accession number: GSE85614).

### Western blotting

Immunoblotting was carried out with rabbit anti‐NCAPG antibodies (1:750; ab56382; Abcam, Cambridge, UK). We used antiglyceraldehyde 3‐phosphate dehydrogenase (GAPDH) antibodies (1:10000, ab8245; Abcam) for an internal loading control. The experimental procedures were performed as described in our past reports 11, 24, 25, 26.

### Plasmid construction and dual‐luciferase reporter assays

A partial wild‐type sequence of the NCAPG 3′‐untranslated region (UTR) or a sequence having a deletion of the *miR‐99a‐3p* target site was inserted into the psiCHECK‐2 vector (C8021; Promega, Madison, WI). The procedures were reported previously 11, 24, 25, 26.

### Immunohistochemistry

Tissue specimens were incubated overnight at 4°C with anti‐NCAPG antibodies (1:150; ab56382; Abcam). The procedures were described previously 11, 15, 24, 25, 26.

### The Cancer Genome Atlas (TCGA) database analyses of PCa

To identify the clinical significance of *NCAPG*, we applied to TCGA database. The gene expression and clinical data were analyzed using cBioportal (http://www.cbioportal.org/) 27. The data were obtained on 30 May 2017.

### Statistical analysis

The relationship between the two groups was analyzed using the Mann–Whitney U test. The relationship of three variables or more was analyzed using Bonferroni‐adjusted Mann–Whitney U tests. The correlation between two groups was evaluated by Spearman's rank test. Survival analyses by Kaplan–Meier method and log‐rank test was performed using JMP software (version 13; SAS Institute Inc., Cary, NC). For all other analyses, Expert StatView (version 5, SAS Institute Inc.) was used.

## Results

### Expression levels of *miR‐99a‐5p* and *miR‐99a‐3p* in PCa specimens and cell lines

**Figure 1:**
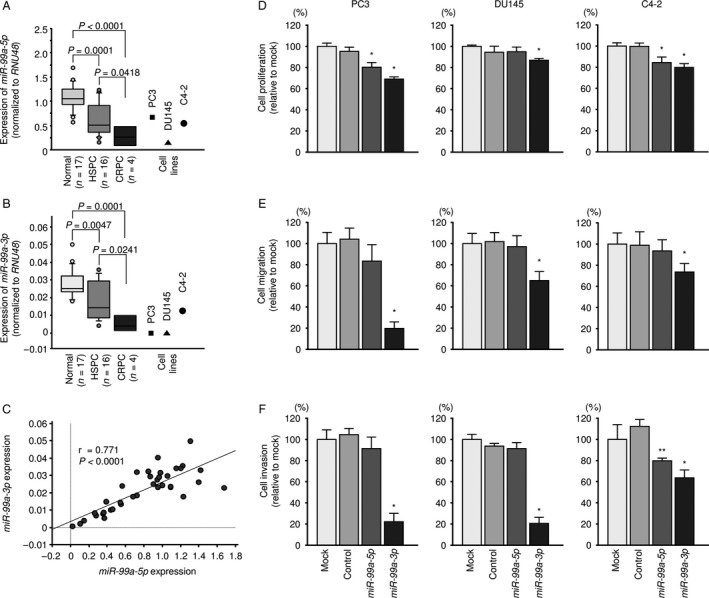
Expression of *miR‐99a‐5p/3p* in clinical prostate specimens and functional analysis of *miR‐99a‐5p/3p* in PCa cell lines. (A) Expression levels of *miR‐99a‐5p* in PCa clinical specimens and cell lines determined using qRT‐PCR. *RNU48* was used as an internal control. (B) Expression levels of *miR‐99a‐3p* in PCa clinical specimens and cell lines. (C) Correlations among the relative expression levels of *miR‐99a‐5p* and *miR‐99a‐3p*. (D‐F) Cell proliferation, migration, and invasion assays in cells transfected with *miR‐99a‐5p/3p*. *P <* *0.0001 and **P <* *0.001.

In human genome, *miR‐99a* is located on chromosome 21q21.1 and the mature sequences of *miR‐99a‐5p* and *miR‐99a‐3p* are 5′‐AACCCGUAGAUCCGAUCUUGUG‐3′and 5′‐CAAGCUCGCUUCUAUGGGUCUG‐3′, respectively (Fig. S1). We validated the expression levels of *miR‐99a‐5p* and *miR‐99a‐3p* in PCa tissues (hormone‐sensitive prostate cancer [HSPC]: *n* = 16, CRPC: *n* = 4), normal tissues (*n* = 17), and PCa cell lines (PC3, DU145, and C4‐2). Table 1 shows the patients’ characteristics. The expression levels of *miR‐99a‐5p* and *miR‐99a‐3p* were markedly lower in PCa and CRPC tissues than in normal tissues (*miR‐99a‐5p*:* P *=* *0.0001 and *P *<* *0.0001, *miR‐99a‐3p*:* P *=* *0.0047 and *P *=* *0.0001; Fig. 1A and B). *miR‐99a‐5p* and *miR‐99a‐3p* were expressed with positive correlation. (*r* = 0.771, *P *<* *0.0001; Fig. 1C). Furthermore, the expression level of pri‐*miR‐99a*, a precursor of *miR‐99a‐5p*/*‐3p*, was also examined and the expression was downregulated in the PCa tissues (Fig. S2).

### Both *miR‐99a‐5p* and *miR‐99a‐3p* bound to Ago2

To verify that both *miR‐99a‐5p* and *miR‐99a‐3p* functioned by incorporation into the RISC, we performed immunoprecipitation with antibodies targeting Ago2 which plays a key role of RISC (Fig. S3A). Quantification of miRNAs bound to Ago2 was detected by PCR methods. The amount of *miR‐99a‐5p* bound to Ago2 was remarkably higher than that in cells transfected with mock, miR‐control, and *miR‐99a‐3*p (*P *<* *0.0001; Fig. S3B). Similarly, the amount of *miR‐99a‐3p* bound to Ago2 was markedly higher than that in cells transfected with mock, miR‐control, and *miR‐99a‐5p* (*P *<* *0.0001; Fig. S3B).

### Effects of restoring *miR‐99a‐5p/3p* on cell proliferation, migration, and invasion activities in PCa cell lines

To confirm the tumor‐suppressive roles of *miR‐99a‐5p* and *miR‐99a‐3p*, we carried out ectopic expression assays by miRNA transfection into PC3, DU145, and C4‐2 cells. According to the results of functional assays, cancer cell proliferation, migration activity, and invasion activity were all remarkably inhibited by transfection with *miR‐99a‐3p* compared with those of mock‐ or miR‐control‐transfected PC3, DU145 C4‐2 cells (*P *<* *0.0001, *P *<* *0.0001, and *P *<* *0.0001, respectively; Fig. 1D–F, S4A and B). Cell proliferation assay was also performed in LNCaP cells, and its ability was suppressed by transfection with *miR‐99a‐3p* (data not shown). In contrast, *miR‐99a‐5p* showed no significant antitumor effects (Fig. 1D‐F).

### Search for putative oncogenes regulated by *miR‐99a‐3p* in PCa cells

**Figure 2:**
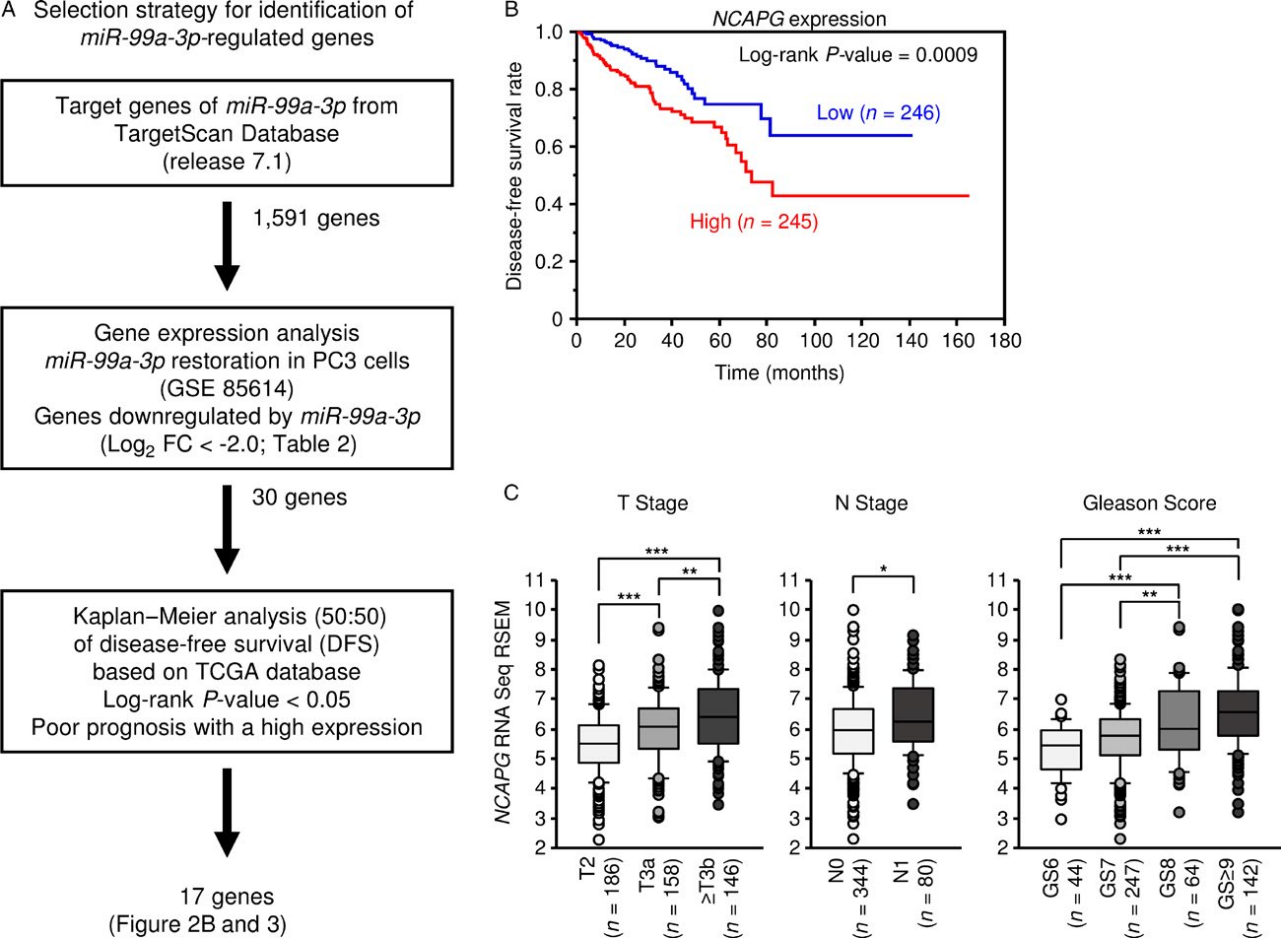
Identification of *miR‐99a‐3p* target genes and relationship between *NCAPG* and clinicopathological factors. (A) Flowchart of the strategy for identification of *miR‐99a‐3p* target genes. (B) Kaplan–Meier patient survival curves for disease‐free survival rates based on *NCAPG* expression in patients with PCa from TCGA database. (C) According to TCGA database, the expression levels of *NCAPG* were significantly increased in cases of advanced T stage, advanced *N* stage, and high Gleason score. *P <* *0.01, **P <* *0.001, and ***P <* *0.0001.

**Figure 3:**
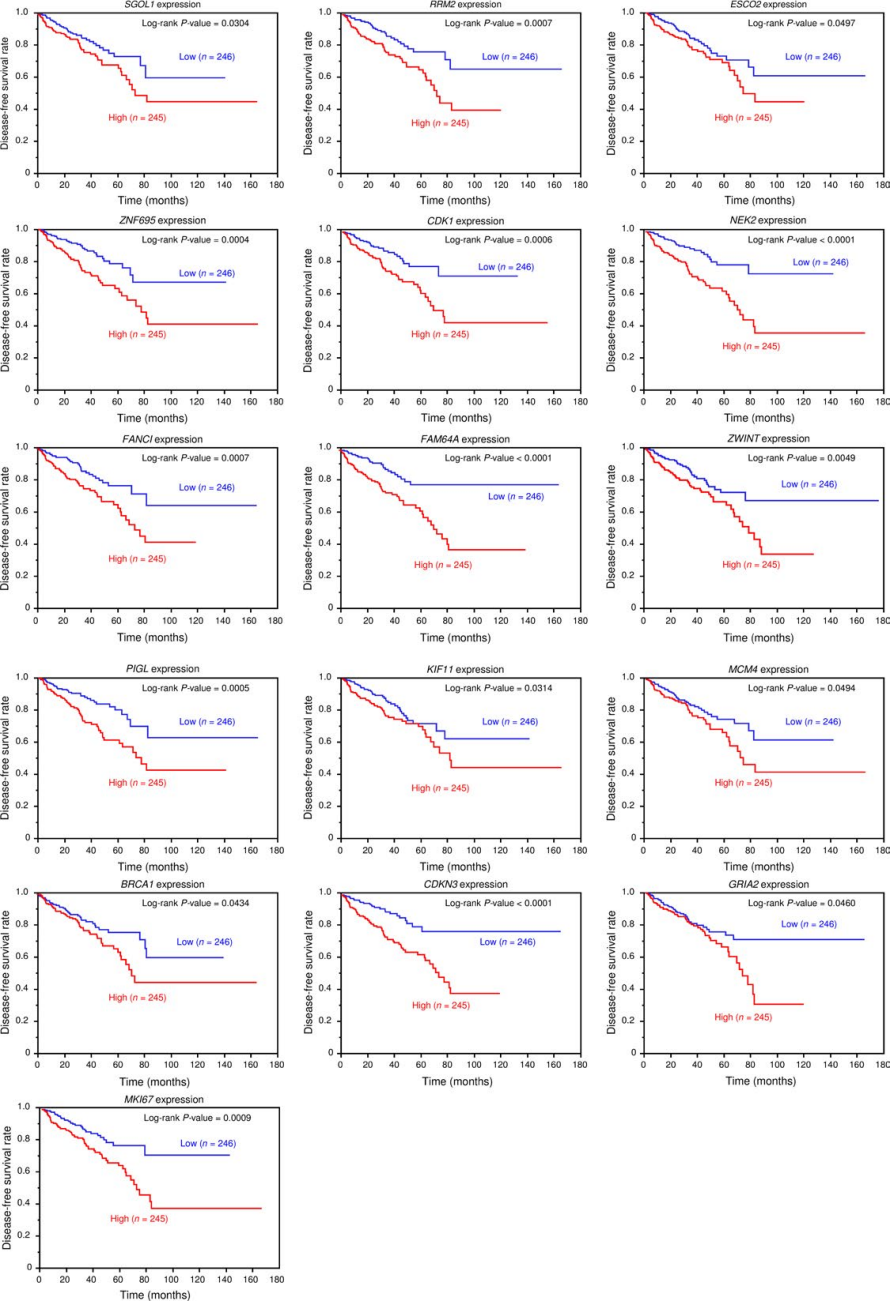
Kaplan–Meier survival curves based on expression of 16 genes, excluding *NCAPG*, in patients with PCa. Kaplan–Meier patient survival curves for disease‐free survival rates based on expression of 16 genes, excluding *NCAPG*, in patients with PCa, according to TCGA database.

We focused on *miR‐99a‐3p*, which showed marked antitumor effects. The selection strategy of *miR‐99a‐3p* target genes is shown in Figure 2A. Initially, we used the TargetScan Human 7.1 database and found that 1591 genes had theoretical target sites for *miR‐99a‐3p* in their 3′‐UTRs. Next, we extracted genes whose expression levels were decreased by transfection with *miR‐99a‐3p* by gene expression analysis (GEO accession number: GSE85614). Genes that were markedly decreased by transfection into PC3 cells with *miR‐99a‐3p* are shown in Table 2 (fold‐change log_2_ < −2.0). In this study, a total of 30 putative oncogenic targets of *miR‐99a‐3p* regulation were identified in PC cells. We investigated further whether it has related to the pathogenesis of PCa and these targets using TCGA database. Among these targets, 17 genes (*NCAPG, SGOL1, RRM2, ESCO2, ZNF695, CDK1, NEK2, FANCI, FAM64A, ZWINT, PIGL, KIF11, MCM4, BRCA1, CDKN3, GRIA2,* and *MKI67*) were involved in PCa pathogenesis, high expression of these genes were significantly associated with disease‐free survival rate (Figs 2B, 3).

**Table 2:** Putative target genes regulated by *miR‐99a‐3p* in PCa cells

Entrez Gene ID	Gene symbol	Gene name	Location	Number of *miR‐99a‐3p* target sites	PC3 *miR‐99a‐3p* transfectant (Log_2_ ratio)
64151	*NCAPG*	Non‐SMC condensin I complex, subunit G	4p15.31	1	−3.87
151648	*SGOL1*	Shugoshin‐like 1 (S. pombe)	3p24.3	1	−3.49
6241	*RRM2*	Ribonucleotide reductase M2	2p25.1	1	−3.39
157570	*ESCO2*	Establishment of sister chromatid cohesion N‐acetyltransferase 2	8p21.1	1	−3.26
57116	*ZNF695*	Zinc finger protein 695	1q44	1	−3.21
113115	*MTFR2*	Mitochondrial fission regulator 2	6q23.3	1	−3.19
983	*CDK1*	Cyclin‐dependent kinase 1	10q21.2	1	−3.03
4751	*NEK2*	NIMA‐related kinase 2	1q32.3	1	−2.82
8693	*GALNT4*	UDP‐N‐acetyl‐alpha‐D‐galactosamine:polypeptide N‐acetylgalactosaminyltransferase 4 (GalNAc‐T4)	12q21.33	2	−2.72
143686	*SESN3*	Sestrin 3	11q21	1	−2.61
55215	*FANCI*	Fanconi anemia, complementation group I	15q26.1	1	−2.57
5557	*PRIM1*	Primase, DNA, polypeptide 1 (49 kDa)	12q13.3	1	−2.56
54478	*FAM64A*	Family with sequence similarity 64, member A	17p13.2	1	−2.56
2218	*FKTN*	Fukutin	9q31.2	2	−2.53
51522	*TMEM14C*	Transmembrane protein 14C	6p24.2	1	−2.50
11130	*ZWINT*	ZW10 interacting kinetochore protein	10q21.1	1	−2.47
9487	*PIGL*	Phosphatidylinositol glycan anchor biosynthesis, class L	17p11.2	1	−2.47
3832	*KIF11*	Kinesin family member 11	10q23.33	1	−2.43
4173	*MCM4*	Minichromosome maintenance complex component 4	8q11.21	1	−2.42
672	*BRCA1*	Breast cancer 1, early onset	17q21.31	1	−2.40
586	*BCAT1*	Branched chain amino‐acid transaminase 1, cytosolic	12p12.1	3	−2.38
1033	*CDKN3*	Cyclin‐dependent kinase inhibitor 3	14q22.2	1	−2.37
79917	*MAGIX*	MAGI family member, X‐linked	Xp11.23	1	−2.36
57082	*CASC5*	Cancer susceptibility candidate 5	15q15.1	1	−2.35
2891	*GRIA2*	Glutamate receptor, ionotropic, AMPA 2	4q32.1	1	−2.30
4288	*MKI67*	Antigen identified by monoclonal antibody Ki‐67	10q26.2	1	−2.25
283487	*LINC00346*	Long intergenic non‐protein coding RNA 346	13q34	1	−2.23
56952	*PRTFDC1*	Phosphoribosyl transferase domain containing 1	10p12.1	1	−2.12
5140	*PDE3B*	Phosphodiesterase 3B, cGMP‐inhibited	11p15.2	1	−2.04
2177	*FANCD2*	Fanconi anemia, complementation group D2	3p25.3	1	−2.01

Finally, we focused on *NCAPG*, which showed the greatest reduction in expression following transfection with *miR‐99a‐3p*.

### Clinical significance of *NCAPG* in PCa

According to TCGA database, *NCAPG* expression levels were closely related to prognosis and clinical stage in patients with PCa. High *NCAPG* expression group had remarkably shorter disease‐free survival (DFS) than that of the low expression group in patients with PCa (*P *=* *0.0009, Fig. 2B). Moreover, the expression levels of *NCAPG* were markedly increased in cases with advanced *T* stage, advanced *N* stage, and high Gleason Score (Fig. 2C). These results indicated that *NCAPG* may affect disease progression and malignancy in PCa.

### *NCAPG* was directly regulated by *miR‐99a‐3p* in PCa cells

**Figure 4:**
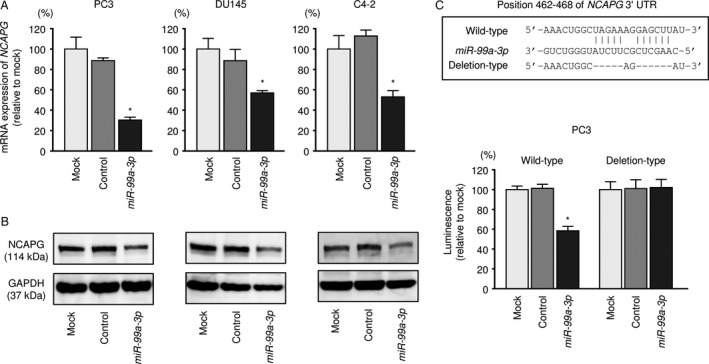
Direct regulation of *NCAPG* by *miR‐99a‐3p* in PCa cells. (A) *NCAPG* mRNA expression was evaluated using qRT‐PCR in PC3, DU145, and C4‐2 cells 48 h after transfection with *miR‐99a‐3p*. *GAPDH* was used as an internal control. *P <* *0.0001. (B) NCAPG protein expression was evaluated by Western blotting in PC3, DU145, and C4‐2 cells 72 h after transfection with *miR‐99a‐3p*. (C) *miR‐99a‐3p* binding sites in the 3′‐UTR of *NCAPG* mRNA. Dual‐luciferase reporter assays in PC3 using vectors encoding a putative *miR‐99a‐3p* target site in the *NCAPG* 3′‐UTR (positions 462–468). Data were normalized by expression ratios of *Renilla*/firefly luciferase activities. *P <* *0.0001.

The expression of *NCAPG* mRNA was significantly decreased by *miR‐99a‐3p* transfection compared to that of mock‐ or miR‐control‐transfected cells (Fig. 4A). Consistent with this, NCAPG protein expression was reduced by *miR‐99a‐3p* transfection (Fig. 4B).

To validate direct binding of *miR‐99a‐3p* in *NCAPG* mRNA, we performed luciferase reporter assays. The TargetScan database predicted that *miR‐99a‐3p* joined at position 462–468 in the 3′‐UTR of *NCAPG*. The luminescence intensity was remarkably reduced by cotransfection with *miR‐99a‐3p* and wild‐type vector of 3′‐UTR of *NCAPG*. In contrast, using the vector in which the target site of *miR‐99a‐3p* was deleted, the luminescence intensity did not change (Fig. 4C).

### Expression of *NCAPG* in PCa clinical specimens

**Figure 5:**
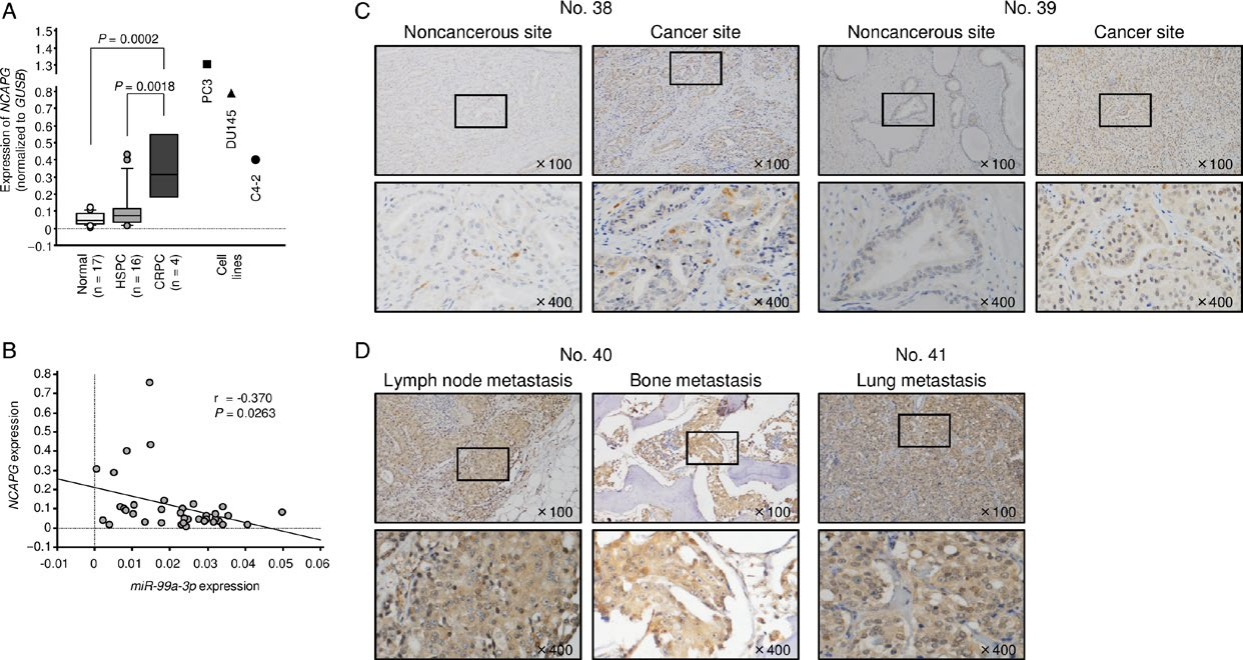
Expression of NCAPG in clinical PCa specimens. (A) Expression levels of *NCAPG* in PCa clinical specimens and cell lines. *GUSB* was used as an internal control. (B) The negative correlation between *miR‐99a‐3p* and *NCAPG*. (C) Immunochemical staining of NCAPG in HSPC specimens. (D) Immunochemical staining of NCAPG in mCRPC specimens.

We evaluated the expression levels of *NCAPG* in PCa tissues (HSPC: *n* = 16, CRPC: *n* = 4), normal tissues (*n* = 17), and PCa cell lines (PC3, DU145, and C4‐2). *NCAPG* was markedly upregulated in CRPC tissues compared with that in normal tissues and HSPC tissues (*P *=* *0.0002, *P *=* *0.0018, respectively; Fig. 5A). Additionally, Spearman's rank test indicated that *miR‐99a‐3p* and *NCAPG* were expressed with negative correlation. (*P *=* *0.0263, *r* = −0.370; Fig. 5B).

Furthermore, to analyze NCAPG protein expression, immunohistochemistry was performed with PCa clinical specimens (Table 1). In CRPC specimens, NCAPG protein was strongly expressed in metastatic tissues from patients with CRPC, compared with non‐PCa or HSPC specimens (Fig. 5C and D).

### Effects of silencing *NCAPG* in PCa cell lines

**Figure 6:**
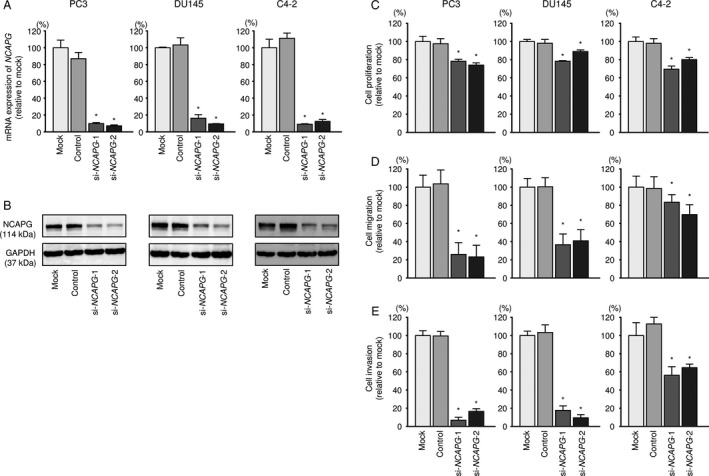
Effects of *NCAPG* silencing in PCa cell lines. (A) *NCAPG* mRNA expression was evaluated using qRT‐PCR analysis of PC3, DU145, and C4‐2 cells 48 h after transfection with si*‐NCAPG*‐1 or si*‐NCAPG*‐2. *GAPDH* was used as an internal control. *P <* *0.0001. (B) NCAPG protein expression was evaluated by Western blot analysis of PC3, DU145, and C4‐2 cells 72 h after transfection with si*‐NCAPG*‐1 or si*‐NCAPG*‐2. GAPDH was used as a loading control. (C‐E) Cell proliferation, migration, and invasion assays following transfection with si‐*NCAPG*‐1 and si‐*NCAPG*‐2. *P <* *0.0001.

We examined the effects of *NCAPG* knockdown in PC3, DU145, and C4‐2 cells using two types of si‐*NCAPG* (si‐*NCAPG‐*1 and si‐*NCAPG*‐2). Two siRNAs effectively downregulated *NCAPG* mRNA and NCAPG protein expression in PC3, DU145, and C4‐2 cells (Fig. 6A and B). Additionally, functional assays indicated that cell proliferation, migration, and invasion were significantly inhibited by knockdown of *NCAPG* in comparison with mock‐ or si‐control‐transfected cells (Fig. 6C‐E, S5A and B). Even in LNCaP cells, cell proliferation assay was performed, and its ability was markedly suppressed by knockdown of *NCAPG* (data not shown).

### Effects of cotransfection with *NCAPG/miR‐99a‐3p* in PC3 cells

**Figure 7:**
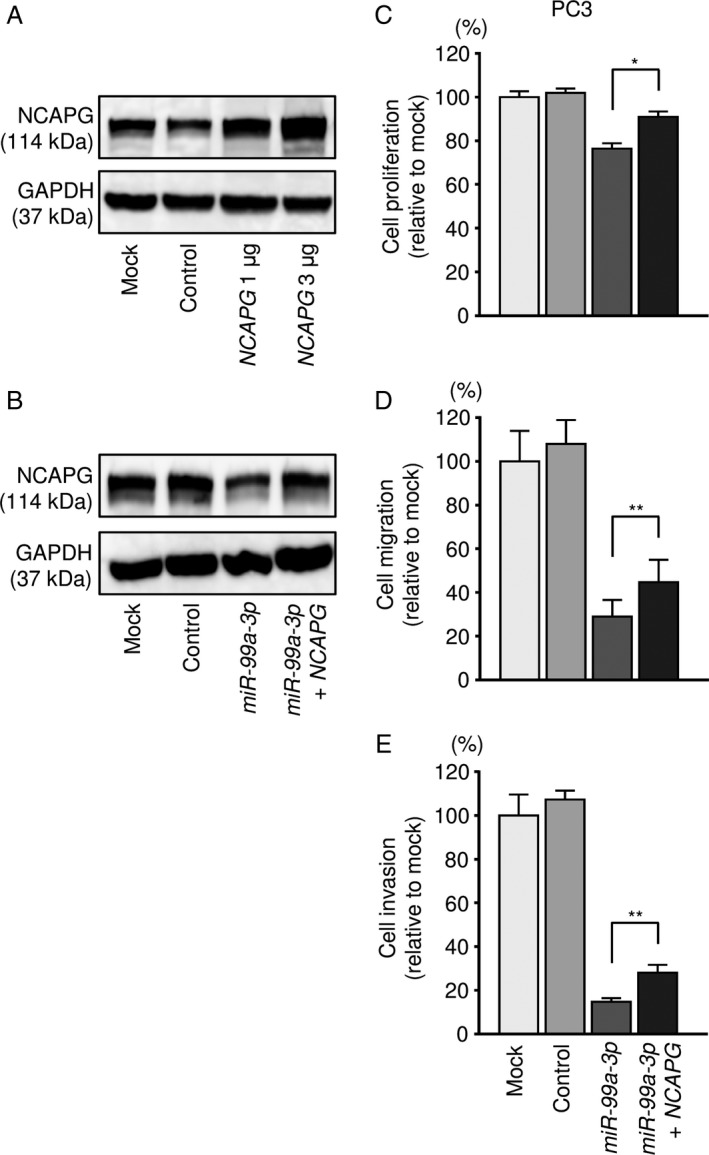
Effects of cotransfection with *NCAPG/miR‐99a‐3p* in PCa cell lines. (A) NCAPG protein expression was evaluated by Western blot analysis of PC3 cells 48 h after forward transfection with the *NCAPG* vector. GAPDH was used as a loading control. (B) NCAPG protein expression was evaluated by Western blot analysis of PC3 cells 72 h after reverse transfection with *miR‐99a‐3p* and 48 h after forward transfection with the *NCAPG* vector. (C) Cell proliferation was determined using XTT assays 72 h after reverse transfection with *miR‐99a‐3p* and 48 h after forward transfection with the *NCAPG* vector. *P <* *0.0001. (D) Cell migration activity was assessed by wound‐healing assays 48 h after reverse transfection with *miR‐99a‐3p* and 24 h after forward transfection with the *NCAPG* vector. **P <* *0.001. (E) Cell invasion activity was characterized by invasion assays 48 h after reverse transfection with *miR‐99a‐3p* and 24 h after forward transfection with the *NCAPG* vector. **P <* *0.001.

We performed *NCAPG* rescue experiments by cotransfection with *NCAPG* and *miR‐99a‐3p* into PC3 cells. Western blot analysis of NCAPG protein expression is shown in Figure 7A and B. According to Western blotting, NCAPG protein levels were recovered by cotransfection with *NCAPG* and *miR‐99a‐3p* in PC3 cells. Moreover, the proliferation, migration, and invasion capacities of PC3 cells were recovered by cotransfection with *NCAPG* and *miR‐99a‐3p* compared with cells transfected with *miR‐99a‐3p* only (Fig. 7C–E, S6A and B). These results indicated that *NCAPG* affected the aggressiveness of PC3 cells.

## Discussion

One of the main challenges in the treatment of CRPC is the control of aggressive and lethal metastatic PCa cells. We believe that identifying genes and pathways involved in metastasis and the acquisition of treatment resistance will lead to the development of new therapeutic strategies. Based on this background, we have identified several antitumor miRNAs, for example, *miR‐1*,* miR‐133a*,* miR‐26a*,* miR‐26b*, the *miR‐29* family, *miR‐205*,* miR‐218*,* miR‐221*,* miR‐222*,* miR‐223*, and *miR‐452*, and showed that these miRNAs target oncogenes 24, 25, 28, 29, 30, 31, 32, 33, 34. Among these oncogenic genes, the extracellular matrix‐related genes laminin *γ*3 (*LAMC3*) and lysyl oxidase‐like 2 (*LOXL2*) were found to be overexpressed in naïve PCa clinical specimens and to enhance cancer cell migration and invasion in PCa cells 30, 31. Moreover, integrin *α*3 (*ITGA3*) and *β*1 (*ITGB1*), heterodimeric transmembrane receptors, were also overexpressed in naïve PCa clinical specimens, and integrin‐mediated oncogenic signaling enhanced cancer cell aggressiveness 25. These molecules are putative therapeutic targets for patients with naïve PCa and CRPC.

In general miRNA biogenesis, guide strand of miRNA is incorporated into RISC (RNA‐induced silencing complex) and acts as a fine‐tuner of expression control. In contrast, passenger strand of miRNA is disassembled and has no function 17, 18, 19. In miRNA biology, miRNA strand selection process is still obscure that which strand become the guide strand or the passenger strand from a miRNA duplex. Recent studies suggested that the thermodynamic character of the duplex seems to play an important role 35. An important feature of the miRNA guide strand is the U‐bias at the 5′end and excess purine, and the passenger strand has a C‐bias at the 5′ end and an excess of pyrimidine 35. The molecular dynamics of miRNA (guide strand and passenger strand) degradation and stabilization in normal and disease cells remain largely unknown.

Despite the previous theory that passenger strands of miRNA have no function, many studies have suggested that some passenger strands have actually functioning in the plant and human cells 36, 37, 38. Our recent studies showed that some passenger strands of miRNAs, for example, *miR‐145‐3p*,* miR‐149‐3p*,* miR‐150‐3p*,* miR‐199a‐3p,* and *miR‐144‐5*p, acted as antitumor miRNAs in several types of cancers 11, 12, 13, 14, 15. *miR‐145‐5p* (guide strand) is known to act as an antitumor miRNA in a variety of cancers through targeting several oncogenes 39, 40, 41. We showed that both strands of pre‐*miR‐145*, that is, *miR‐145‐5p* and *miR‐145‐3p*, were significantly downregulated in CRPC specimens compared with those in naïve PCa or non‐PCa specimens 15. Our data demonstrated that *miR‐145‐3p* (passenger strand) had stronger antitumor effects than *miR‐145‐5p* (guide strand) in PCa cells 15. We also confirmed the antitumor effects of *miR‐145‐3p* in bladder and lung and cancers 42, 43. More recently, we showed that both *miR‐150‐5p* (guide strand) and *miR‐150‐3p* (passenger strand) acted as antitumor miRNAs through targeting SPARC/osteonectin and cwcv and kazal‐like domains proteoglycan 1 (*SPOCK1*) in naïve PCa and CRPC cells 11. The involvement of passenger strand miRNAs in cellular processes regulation is a new conception in RNA research.

In this study, we focused on *miR‐99a‐5p* whose expression was significantly downregulated in our miRNA signature of metastatic CRPC and investigated the functional roles including passenger strand *miR‐99a‐3p* in PCa cells. As the results, we indicated that *miR‐99a‐3p* has potent antitumor effects in PCa cells. The expression levels of the two miRNAs, *miR‐99a‐5p* and *miR‐99a‐3p,* were obviously different in clinical specimens and cancer cell lines. We do not see any clear answer as to why this kind of difference will arise. This challenge is an important issue for miRNA research. In addition, a more detailed study on the concentration of miRNAs to be transfected into cancer cells and antitumor effects will be necessary.

The *miR‐99a‐5p* (guide strand) has been reported to have tumor‐suppressive roles in various types of cancers, including PCa 20, 21, 22, 23, 44. In nonsmall‐cell lung cancer, *miR‐99a‐5p* was reported to suppress cancer cell proliferation and metastasis by controlling the AKT1 signaling pathway and insulin‐like growth factor‐1 receptor, which could also serve as a diagnostic biomarker 23, 44. Additionally, several recent reports demonstrated the antitumor effects of *miR‐99a‐5p* on mammalian target of rapamycin (mTOR) regulation 20, 21, 22. For example, *miR‐99a‐5p* directly regulates the mTOR pathway to induce G_1_‐phase cell cycle arrest and suppress tumorigenicity in renal cell carcinoma 21. Additionally, in PCa, the *miR‐99* family, including *miR‐99a‐5p*, directly targets the chromatin‐remodeling factors *SMARCA5* and *SMARCD1* and the growth regulatory kinase mTOR, suppresses the expression of PSA, and blocks PCa cell proliferation 45. Furthermore, inhibition of the *miR‐99a/let‐7c/miR‐125b‐2* miRNA cluster promotes the induction of several androgen‐induced genes and stimulates the initiation and progression of PCa 46.

In contrast, the passenger strand *miR‐99a‐3p* has been reported as a diagnostic marker of the chemotherapy response in patients with advanced colorectal cancer 47; however, there are no reports examining the functional significance of *miR‐99a‐3p* in cancer cells. Our previous studies of miRNA signatures showed that *miR‐99a‐3p* was significantly downregulated in bladder cancer, renal cell carcinoma, and head and neck squamous cell carcinoma, suggesting *miR‐99a‐3p* has antitumor roles in these cancers 48, 49, 50. Moreover, TCGA database revealed that low expression of *miR‐99a‐3p* was significantly associated with poor prognosis in head and neck cancer and lung adenocarcinoma (Fig. S7). This is the first report demonstrating that *miR‐99‐3p* may function as an antitumor miRNA in naïve PCa and CRPC cells.

Unique nature of miRNA, single miRNA controls vast number of genes in normal and cancer cells. We performed gene expression analyses and in silico database search to identify *miR‐99a‐3p* regulated oncogenic genes in PCa cells. Interestingly, a large number of cohort analyses by TCGA database showed several targets were deeply involved PCa pathogenesis. These genes might be important tools for elucidating the molecular pathogenesis of PCa and CRPC.

In this study, by focusing on *miR‐99a‐3p*, which had not been well studied in previous reports, we found that *NCAPG* was directly regulated by *miR‐99a‐3p* in PCa cells. Overexpression of NCAPG was observed in CRPC clinical specimens, and its expression was found to be essential for PCa pathogenesis, as demonstrated by analysis of TCGA database. Interestingly, our previous study indicated that *NCAPG* was regulated by *miR‐145‐3p* in PCa cells 15. Thus, *NCAPG* is a candidate gene controlled by multiple antitumor miRNAs in CRPC, and its function in the pathogenesis of PCa may be important. However, the cancer‐promoting functions of this molecule are still not well known.

*NCAPG* is involved in mitotic chromosome condensation and is related to the cell cycle. Mitotic chromosome condensation is an essential cellular property of all proliferating cells and results in reconstitution of chromosomes into rod‐like mitotic chromosomes, ensuring separation of sister chromatids during cell division. In vertebrates, there are two types of condensin complexes, type I and II complexes, both of which contain nonstructural maintenance of chromosomes (non‐SMC) regulatory subunits. Defects in one of the subunits cause incomplete chromosome condensation 51, 52. NCAPG exists in the condensin I complex and is associated with proper segregation of sister chromatids in the condensation and fission of mitotic chromosomes 53. Previous studies showed that *NCAPG* was involved in the cell cycle and had cancer‐promoting functions in several types of cancers 54, 55. A recent study showed that knockdown of *NCAPG* induced apoptosis, reduced cancer cell survival, and suppressed the epithelial–mesenchymal transition (EMT) in cancer cells via upregulation of Bax, cleaved caspase‐3, and E‐cadherin and downregulation of cyclin A1, CDK2, Bcl‐2, N‐cadherin, and HOXB9 in hepatocellular carcinoma 55. Our present data showed that aberrant expression of *NCAPG* enhanced PCa cell aggressiveness. Thus, these data suggested that *NCAPG* had clinical significance in PCa pathogenesis and could have applications as a therapeutic target in CRPC.

In conclusion, both strands of pre‐*miR‐99a*, that is, *miR‐99a‐5p* and *miR‐99a‐3p*, were significantly reduced in naïve PCa and CRPC clinical specimens. The passenger strand, *miR‐99a‐3p*, had potent antitumor effects via targeting of the oncogene *NCAPG* in PCa cells. *NCAPG* was markedly elevated in CRPC and was involved in CRPC pathogenesis, suggesting that *NCAPG* could have applications as a therapeutic target in CRPC. The involvement of passenger strand miRNAs in cancer cells is novel concept of naïve PCa and CRPC pathogenesis.

## Conflict of Interest

The authors declare no conflict of interests.

## Supporting information


**Figure S1**. Schematic representation of the chromosomal location of human *miR‐99a*.

**Figure S2.** Expression levels of pri‐*miR‐99a* in PCa clinical specimens and cell lines.

**Figure S3.** Both strands of *miR‐99a‐5p* and *miR‐99a‐3p* incorporated into the RISC.

**Figure S4**. Phase micrographs of wound healing and invasion assays following transfection with *miR‐99a‐5p/3p* in PCa cell lines.

**Figure S5**. Phase micrographs of wound healing and invasion assays following transfection with si‐*NCAPG* in PCa cell lines.

**Figure S6**. Phase micrographs of wound healing and invasion assays following cotransfection with *NCAPG/miR‐99a‐3p* in PC3 cells.

**Figure S7.** Kaplan‐Meier survival curves based on miR‐99a‐3p expression in patients with Head and Neck squamous cell carcinoma and Lung adenocarcinoma.

**Table S1.** Product numbers of reagents**.**
