# Supplementary figures and images for: Regulation of NCAPG by miR‐99a‐3p (passenger strand) inhibits cancer cell aggressiveness and is involved in CRPC
Source: Cancer Med. 2018 Apr 2;7(5):1988–2002. doi: 10.1002/cam4.1455 (PMC5943442; doi:10.1002/cam4.1455)

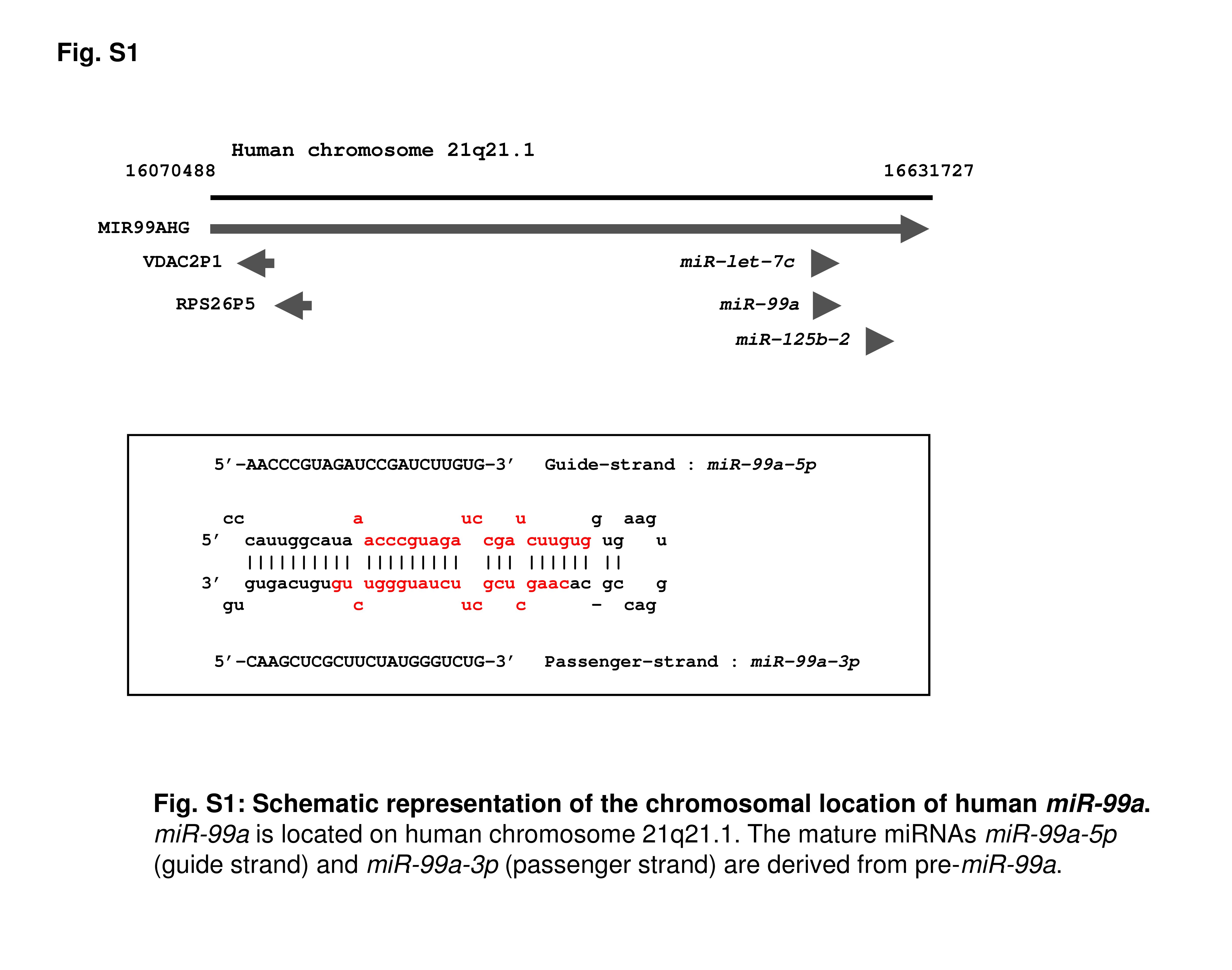

Supplement: Supplementary file 1 — Figure S1. Schematic representation of the chromosomal location of human miR‐99a. [file CAM4-7-1988-s001.tif]

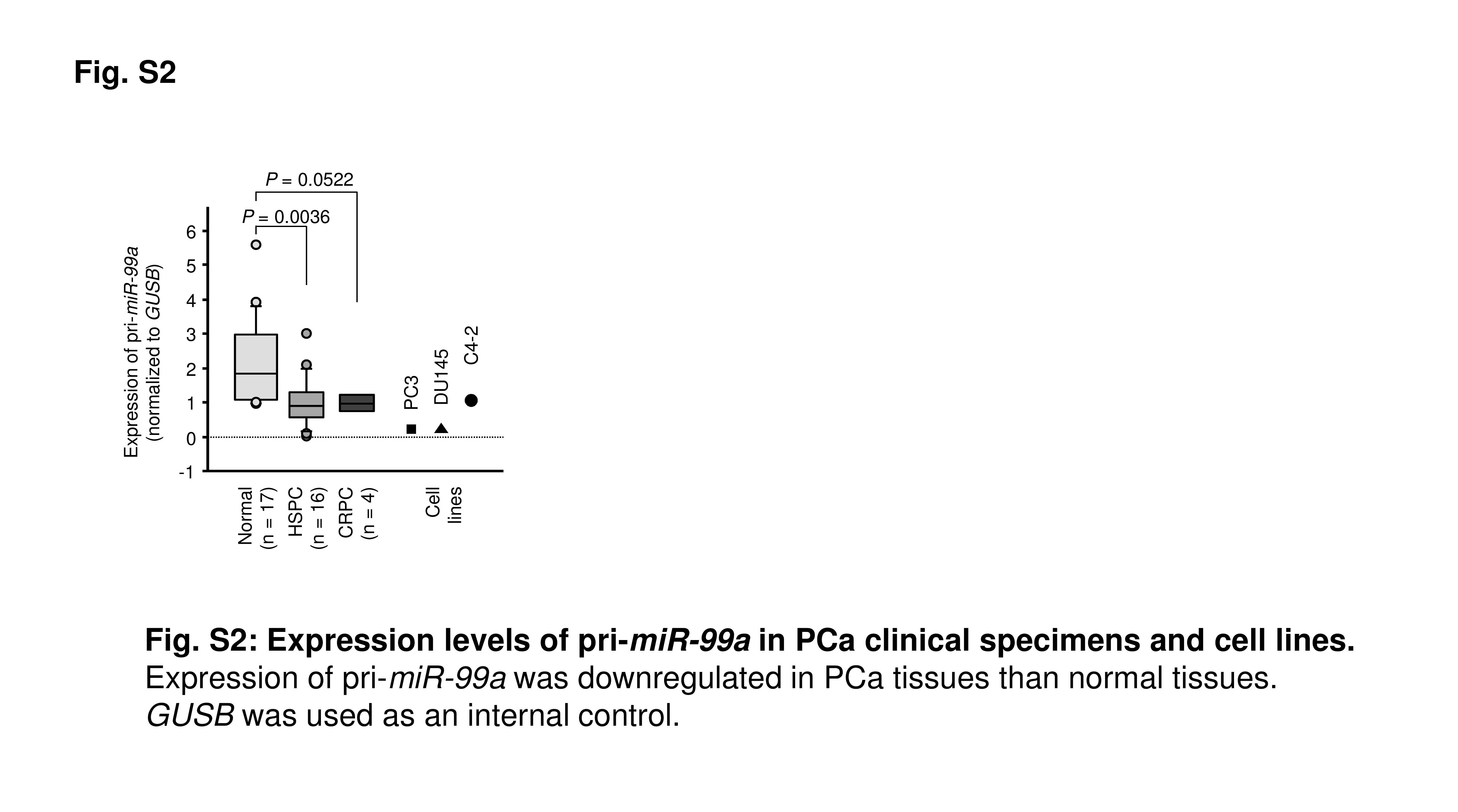

Supplement: Supplementary file 2 — Figure S2. Expression levels of pri‐miR‐99a in PCa clinical specimens and cell lines. [file CAM4-7-1988-s002.tif]

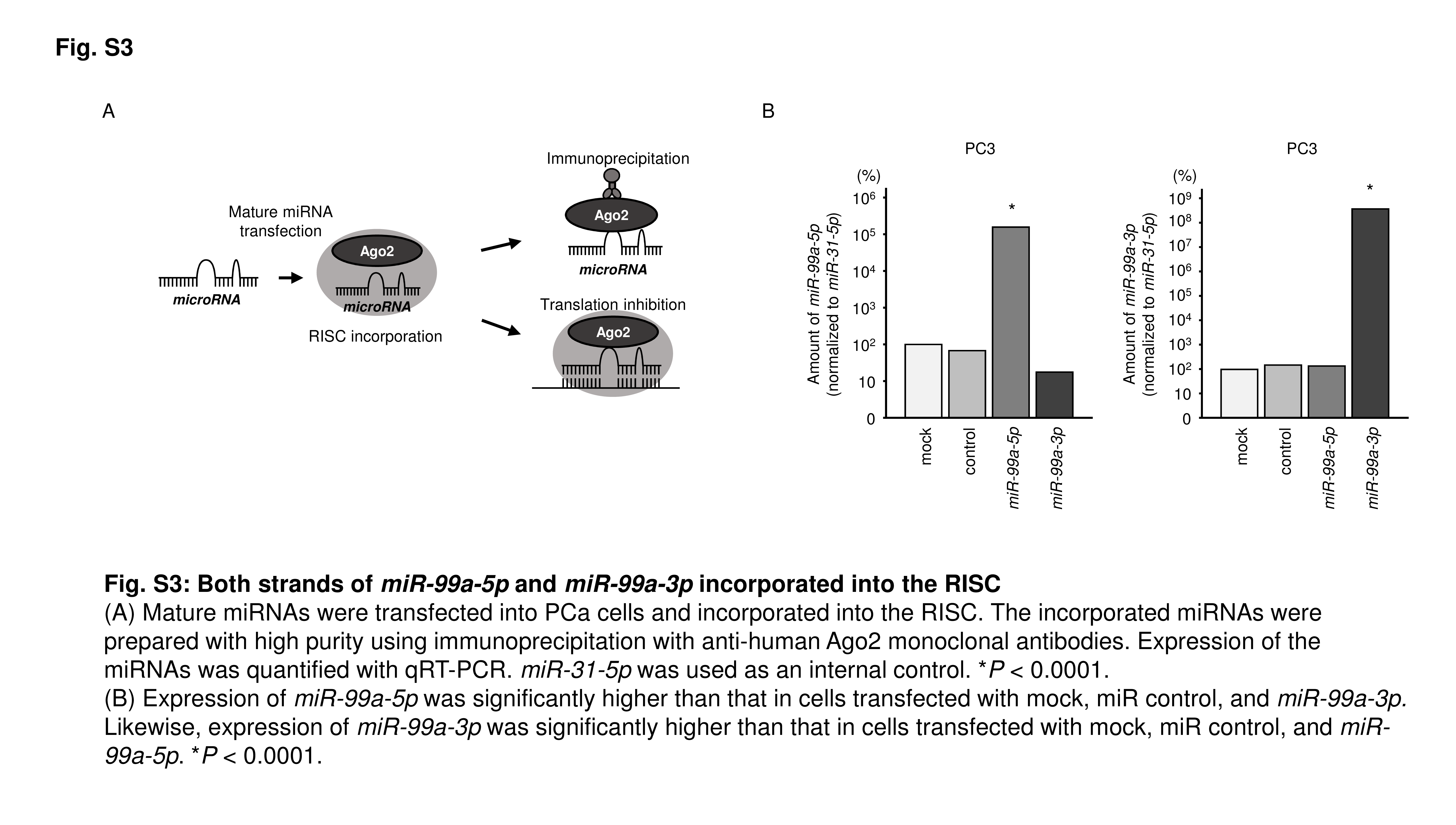

Supplement: Supplementary file 3 — Figure S3. Both strands of miR‐99a‐5p and miR‐99a‐3p incorporated into the RISC. [file CAM4-7-1988-s003.tif]

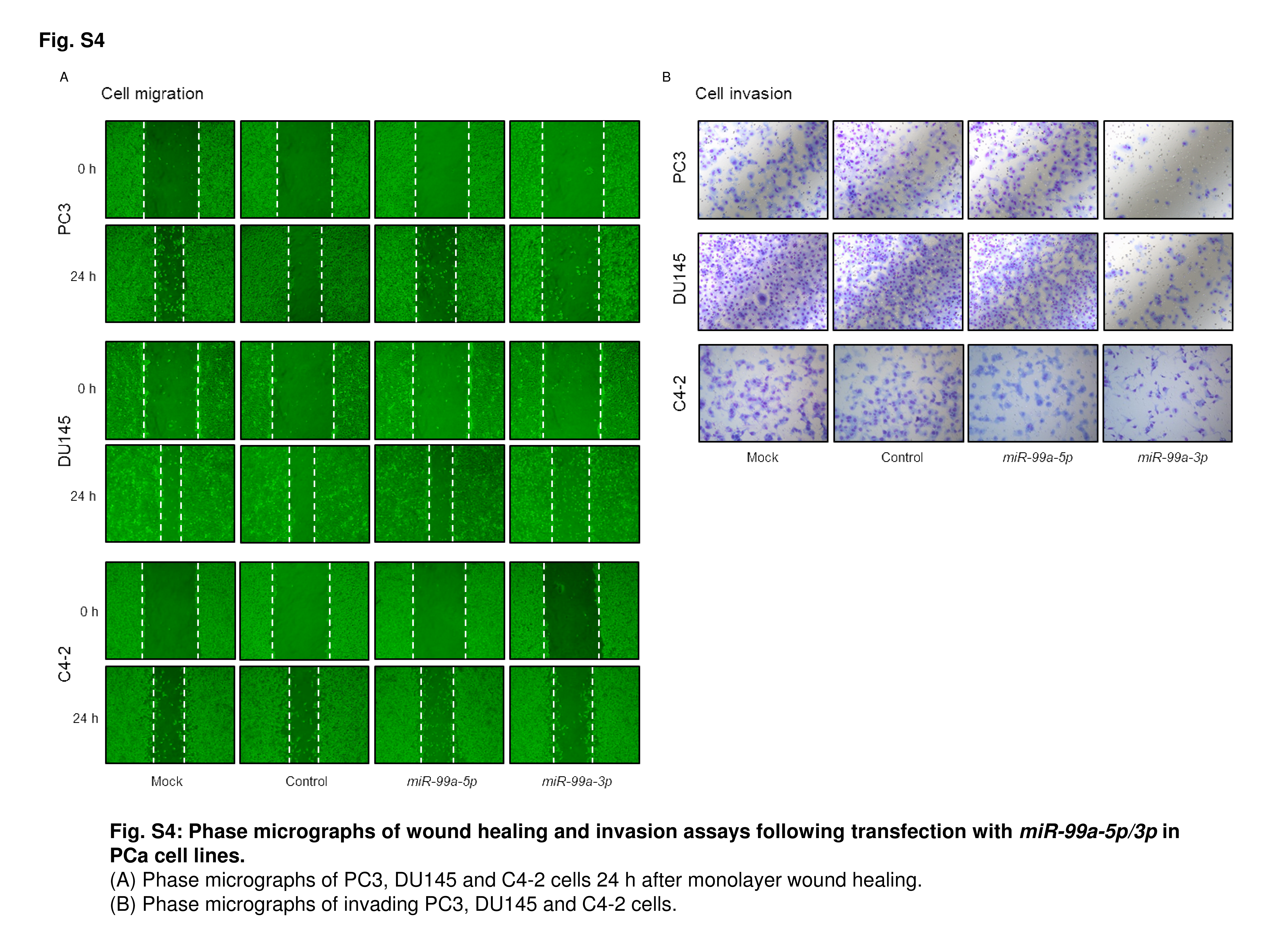

Supplement: Supplementary file 4 — Figure S4. Phase micrographs of wound healing and invasion assays following transfection with miR‐99a‐5p/3p in PCa cell lines. [file CAM4-7-1988-s004.tif]

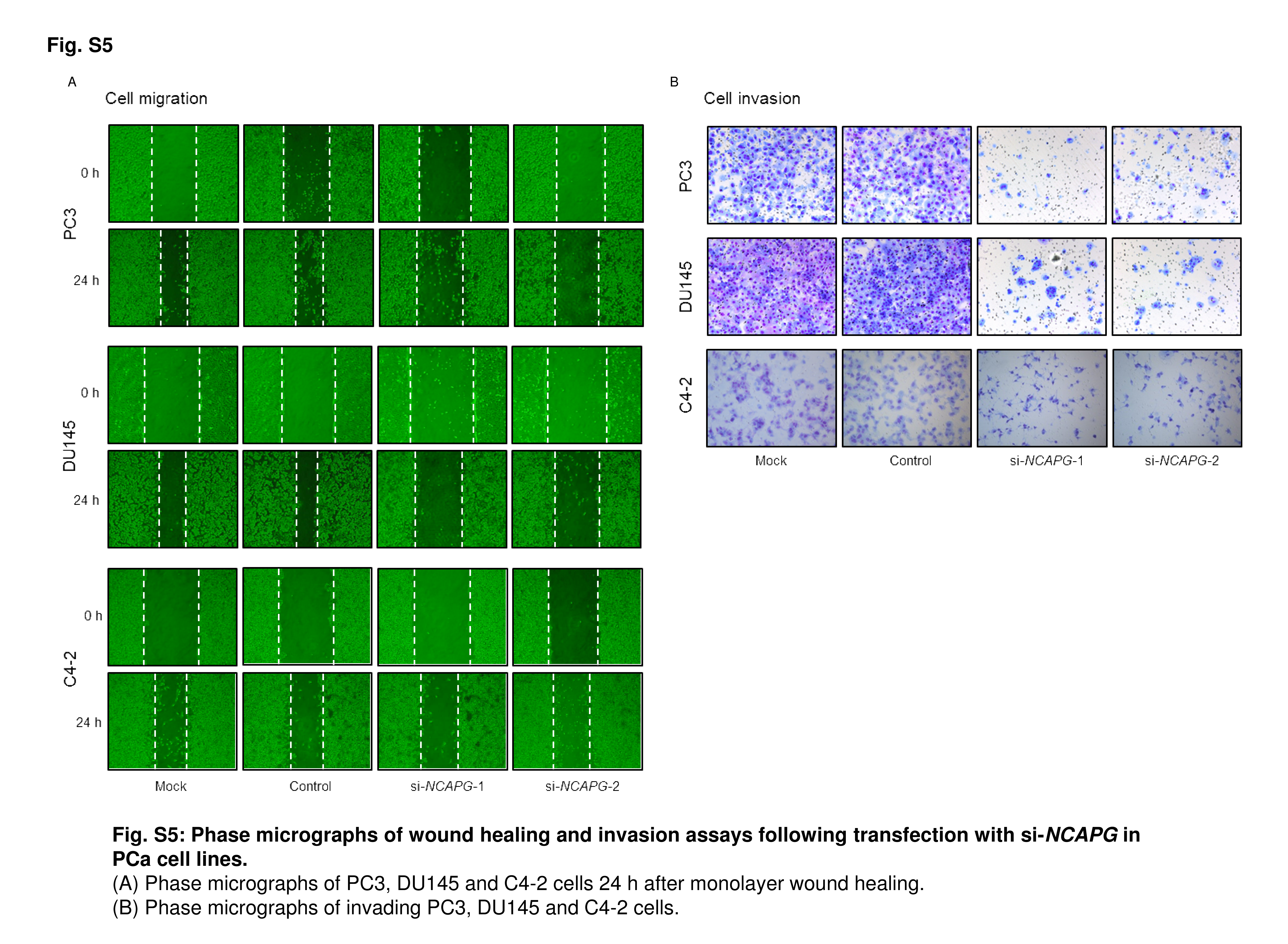

Supplement: Supplementary file 5 — Figure S5. Phase micrographs of wound healing and invasion assays following transfection with si‐NCAPG in PCa cell lines. [file CAM4-7-1988-s005.tif]

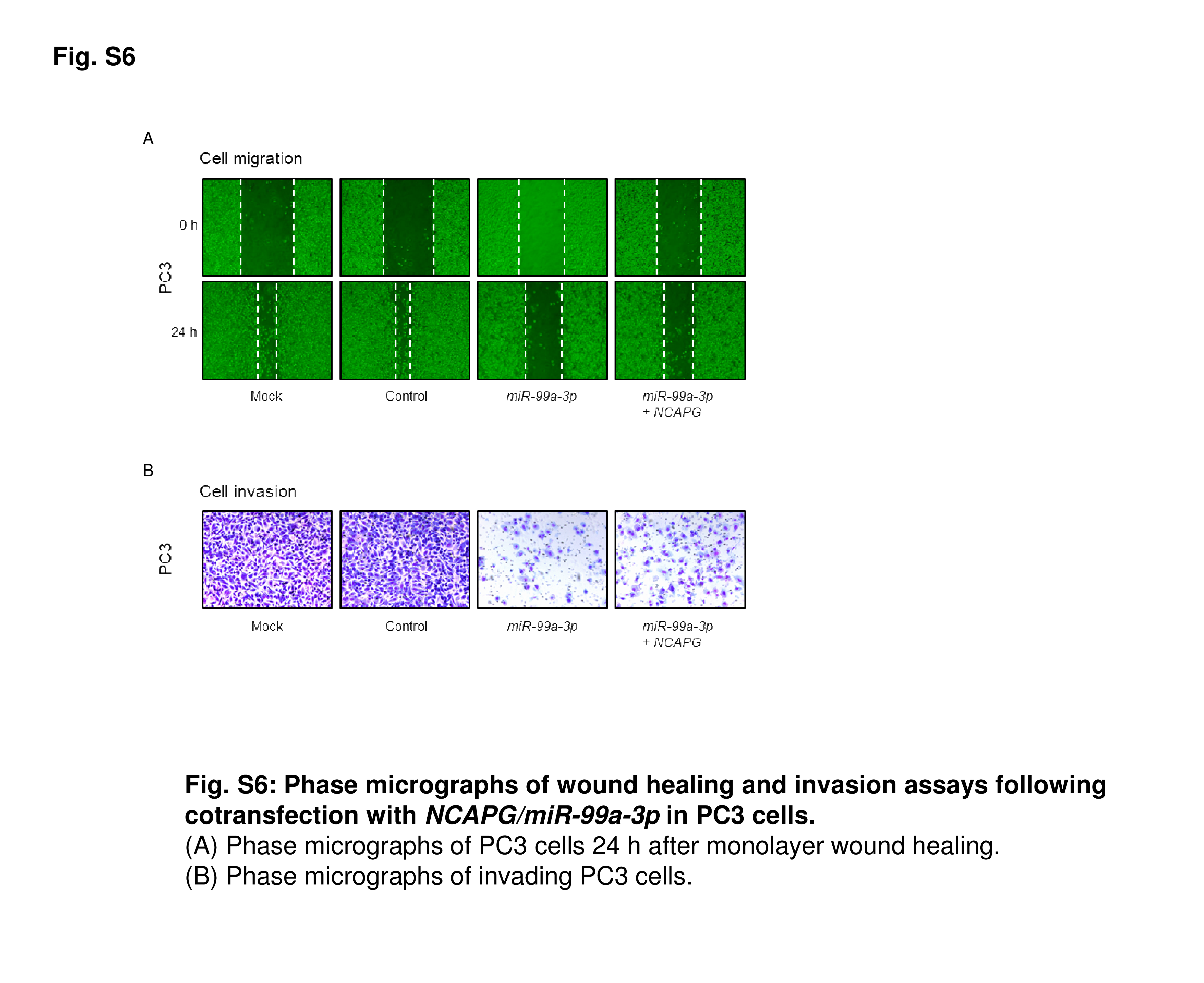

Supplement: Supplementary file 6 — Figure S6. Phase micrographs of wound healing and invasion assays following cotransfection with NCAPG/miR‐99a‐3p in PC3 cells. [file CAM4-7-1988-s006.tif]

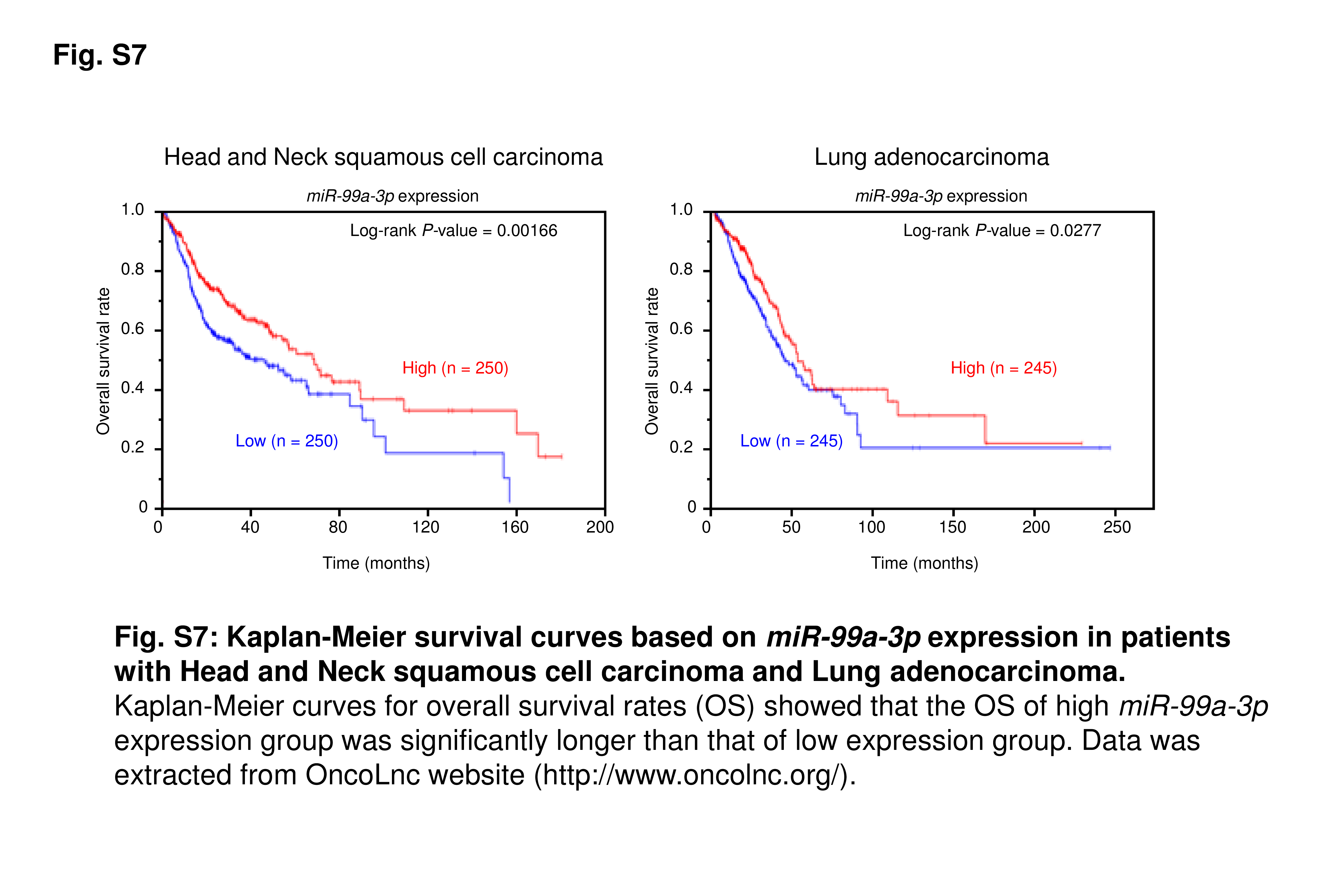

Supplement: Supplementary file 7 — Figure S7. Kaplan‐Meier survival curves based on miR‐99a‐3p expression in patients with Head and Neck squamous cell carcinoma and Lung adenocarcinoma. [file CAM4-7-1988-s007.tif]
